# *Paradiplozoon yunnanensis* n. sp. (Monogenea, Diplozoidae) from *Sikukia gudgeri* (Cyprinidae, Barbinae) in southwest China

**DOI:** 10.1051/parasite/2018047

**Published:** 2018-09-07

**Authors:** Li-xian Fan, Fei-yan Meng, Jun-ping Bai, Wei-jiang Xu, Xu Wang

**Affiliations:** 1 School of Life Sciences of Yunnan Normal University Kunming China; 2 Engineering Research Center of Sustainable Development and Utilization of Biomass Energy, Ministry of Education Kunming China

**Keywords:** Diplozoidae, *Paradiplozoon*, ITS2, *Sikukia gudgeri*, China

## Abstract

*Paradiplozoon yunnanensis* n. sp. (Monogenea, Diplozoidae) is described from the gills of *Sikukia gudgeri* Smith, 1931 (Cyprinidae) collected from Jinghong Basin, a tributary of the international Lancang-Mekong River. This is the first diplozoid species from *S. gudgeri* and its description increases the number of *Paradiplozoon* species recorded in China to 25. The new species is distinguished from congeners by a combination of morphological and molecular features. The anterior end of the median plate is thickened in the marginal area and a narrow rectangular trapeze spur connects to the anterior jaw through two separate anterior joining sclerites. The posterior end of the median plate sclerite is invaginated with a smooth strip-shaped posterior joining sclerite. Comparison of a newly obtained sequence of rRNA ITS2 with 18 other congeneric sequences from GenBank provides support for separation of the new species.

## Abstract (fr)

*Paradiplozoon yunnanensis* n. sp. (Monogenea, Diplozoidae) est décrit des branchies de *Sikukia gudgeri* Smith, 1931 (Cyprinidae) collecté dans le bassin de Jinghong, un affluent de la rivière internationale Lancang-Mékong. C’est la première espèce de Diplozoidae de *S. gudgeri* et sa description amène le nombre d’espèces de *Paradiplozoon* de Chine à 25. La nouvelle espèce se distingue de ses congénères par une combinaison de caractéristiques morphologiques et moléculaires. L’extrémité antérieure de la plaque médiane est épaissie dans la zone marginale et un étroit éperon trapézoïdal rectangulaire se connecte à la mâchoire antérieure par l’intermédiaire de deux sclérites antérieurs séparés. L’extrémité postérieure de la plaque sclérite médiane est invaginée avec un sclérite postérieur lisse en forme de bande. La comparaison de la séquence d’ARNr ITS2 nouvellement obtenue avec 18 autres séquences congénères de GenBank soutient la séparation de la nouvelle espèce.

## Introduction

Diplozoid monogeneans (Diplozoidae Palombi, 1949) are common blood-feeding ectoparasites on the gills of cyprinid fishes. The Diplozoidae includes five genera in China [32]: *Diplozoon* Nordmann, 1832; *Paradiplozoon* Achmerov, 1974; *Inustiatus* Khotenovsky, 1978; *Sindiplozoon* Khotenovsky, 1981 and *Eudiplozoon* Khotenovsky, 1985. Seventy diplozoid species have been reported globally. Since the first diplozoid species was recorded from China in 1973, a total of 34 species belonging to 5 genera (24 in *Paradiplozoon*, 6 in *Sindiplozoon*, 2 in *Inustiatus*, 1 in *Diplozoon* and 1 in *Eudiplozoon*), have been recorded in China [32, 10, 4, 29, 30].

The lifecycle of diplozoids is direct and includes a free-swimming oncomiracidia and a post-oncomiracidial stage known as a diporpa. Two larvae (diporpa) pair and subsequently fuse permanently into the typical X-shaped body arrangement, a characteristic unique to the Diplozoidae [17]. Diplozoids have developed a sclerotized attachment apparatus for successful attachment to gills. This apparatus includes central hooks and clamps located on the haptor. The central hooks are used by the oncomiracidia, while the clamps are used by diporpa and adults to attach to the gills of the host [16, 17]. These sclerotized structures are considered to be reliable features for species identification via morphological studies [15, 21, 22].

The taxonomy of monogeneans is generally based on the morphology of sclerotized structures of their haptor [31]. In diplozoids, the sclerotized parts of the haptor, in particularly the clamps and central hook, supply the main morphological characters for species discrimination [12, 15, 19, 20, 25, 27]. The identification of species within *Paradiplozoon* is mainly based on the length of the central hook and the shape of certain clamp sclerites [15]. Combination of the shape of the trapeze spur and anterior joining sclerites could lead to accurate discrimination of species [22]. However, the measurements of these morphological characteristics may present high interspecific similarity [5] and show extensive variation with different hosts, water temperatures or geographical origin of parasites [21]. In *Paradiplozoon homoion* Bychowsky & Nagibina, 1959, it was found that specimens parasitizing larger fish such as *Rutilus rutilus* have larger clamps than those from smaller fish such as *Phoxinus phoxinus* [21]. Furthermore, the size and shape of these sclerotized structures may easily be misinterpreted and change according to fixation and preparation [11]. The application of molecular systematics to Platyhelminthes (Cestoda, Digenea and Monogenea) has provided new insights into the interrelationship between species [23]. Due to the complicated identification of several groups of monogenean parasites, molecular markers based on species-specific variability have been designed and shown to be useful for identification of Monogenea. Of these molecular markers, the ITS2 region has been used to distinguish congeneric diplozoids at the species level and provided species identification similar to that made by using morphological structures [1]. Combination of morphological parameters and molecular markers is considered to be a useful approach in the study of various diplozoid taxa [3, 9, 22, 25, 28].

This study presents the identification of a new species from the gills of *Sikukia gudgeri* Smith, 1931 (Cyprinidae: Barbinae) combining morphological and molecular approaches. This study increases the number of species of the genus *Paradiplozoon* recorded in China to 25.

## Materials and methods

### Sample collection

In October 2012, November 2013 and May 2015, a total of 52 *S. gudgeri* were collected from Jinghong Basin, a main tributary of the international Lancang-Mekong River in southwest China. The taxonomic status of fish specimens was determined according to the checklist of fishes and fauna of Yunnan Province [6]. Fish were euthanized by severing the spinal cord posterior to the skull with a single cut [6, 7]. Gills were removed and examined under an Olympus SZX7 dissecting microscope (Olympus, Japan). Specimens were washed in double-distilled water before being preserved in either 70% or 100% ethanol for morphological and molecular research, respectively.

### Morphological analysis

Diplozoid specimens were stained with acetocarmine, differentiated using HCl in 70% ethanol, dehydrated through a graded ethanol series, cleared in clove oil or xylene and mounted in Canada Balsam. Parasites were identified according to Khotenovsky [15] and Wu et al. [32], and were examined and measured using an Olympus CX41 light microscope (Olympus, Japan) and photographed using an Olympus BX53 compound microscope equipped with differential interference contrast (DIC). A digital image analysis system (TCapture Imaging Application 4.2) was used for morphometric analysis and drawing diagnostic morphometric attachment apparatus. All sclerite parts of the attachment apparatus were named according to Jirsová et al. [13] and Al-Nasiri and Balbuena [2]. Measurements of the main characteristics of specimens are given in the results section as a mean value, with the number (*n*) and range of measurements provided in parentheses.

### Molecular analysis

Ethanol-fixed parasites were soaked for 1 day in TE buffer (pH 8.0), and genomic DNA was extracted from each individual specimen using an E.Z.N.A.^®^FFPE DNA Kit (OMEGA Bio–Tek, Inc., Norcross, Georgia, USA) according to the manufacturer’s instructions. According to Matějusová et al., universal eukaryotic primers, D (5′–GGCTYRYGGNGTCGATGAAGAACGCAG–3′) and B1 (5′–GCCGGATCCGAATCCTGGTTAGTTTCTTTTCC T–3′), were used to amplify the ITS2 rDNA [20]. PCR amplification was performed in a 50 μL volume containing 2 μL of DNA template, 19 μL of reaction mixture (dNTP, 10× buffer, Taq polymerase), 2 μL of each primer and 25 μL of double-distilled water, under the following conditions: 10 min at 90 °C; 30 cycles of 30 s at 95 °C, 30 cycles of 30 s at 55 °C, and 75 s at 72 °C; and a final extension of 10 min at 72 °C. PCR products were detected on ethidium bromide-stained 1% agarose gels. Amplicons were analysed using an ABI–3730XL Genetic Analyzer under the appropriate module (Applied by Beijing Connaught Genome Research Center Co. Ltd., Beijing). The sequences obtained were analysed using DNAMAN 7.0 and Sequencher 5.0 (Gene Codes Corp.) software before being deposited in GenBank (MF775370). The accession numbers of diplozoid species retrieved from GenBank and used in the alignment, their host species, and their sampling localities are given in Table 1. The outgroup *Eudiplozoon nipponicum* was used to root resultant phylogenies [25]. Base composition data, parsimony and nucleotide substitutions between pairwise distances (Kimura 2–parameter) were estimated using MEGA 6.0 and BLAST from NCBI. The robustness of topologies was assessed by 1000 bootstrap replicates.

**Table 1.** List of diplozoid species used for genetic comparison and phylogenetic research with *Paradiplozoon yunnanensis* n. sp., including their host species, locality, GenBank accession number and sequence length.

Parasite species	Host species	Locality	GenBank accession number	Length (bp)
1. *Eudiplozoon nipponicum* (Goto, 1891)	*Cyprinus carpio* (Linnaeus, 1758)	Morava River, Czech Republic	AJ300710	755
2. *E. nipponicum* (Goto, 1891)	*Cyprinus carpio* (Linnaeus, 1758)	France	AF369758	977
3. *E. nipponicum* (Goto, 1891)	*Cyprinus carpio* (Linnaeus, 1758)	Irtysh River, China	KP340975	772
4. *E. nipponicum* (Goto, 1891)	*Cyprinus carpio* (Linnaeus, 1758)	Tangxun Lake, China	DQ098895	805
5. *E. nipponicum* (Goto, 1891)	*Cyprinus carpio* (Linnaeus, 1758)	Danjiangkou Reservoir, China	DQ098897	804
6. *Paradiplozoon vaalense* (Dos Santos, 2013)	*Labeo umbratus* (Smith, 1841)	Vaal River, South Africa	HG423142	700
7. *P. krugerense* (Dos Santos, 2016)	*Labeo rosae* (Steindachner, 1894) & *Labeo congoro* (Peters, 1852)	Kruger National Park, South Africa	LT574865	757
8. *P. ichthyoxanthon* (Avenant-Oldewage, 2013)	*Labeobarbus aeneus* (Burchell, 1822)	Vaal River, South Africa	HF566124	733
9. *P. skrjabini* (Achmerov, 1974; Khotenovsky,1985)	*Rhynchocypris lagowskii* (Dybowski, 1869)	Narva River, Russia	LC050529	683
10. *P. nagibinae* (Gläser, 1965)	*Abramis ballerus* (Linnaeus, 1758)	Kyjovka River, Czech Republic	AJ563371	761
11. *P. pavlovskii* (Bychowsky & Nagibina, 1959)	*Abramis aspius* (Linnaeus, 1758)	Morava River, Czech Republic	AJ300714	770
12. *P. sapae* (Reichenbach-Klinke, 1961)	*Abramis sapa* (Pallas, 1814)	Morava River, Czech Republic	AJ300713	769
13. *P. bliccae* (Reichenbach-Klinke, 1961)	*Bliccae bjoerkna* (Linnaeus, 1758)	Morava River, Czech Republic	AJ300712	736
14. *P. megan* (Bychowsky & Nagibina, 1959)	*Leuciscus cephalus* (Linnaeus, 1758)	Morava River, Czech Republic	AJ300711	774
15. *P. bingolensis* (Civáňová *et al*., 2013)	*Garra rufa* (Heckel, 1843)	Murat River, Turkey	HE653910	725
16. *P. gracile* (Reichenbach Klinke, 1961)	*Gobio acutipinnatus* (Men’schikov, 1939)	Irtysh River (Chinese section), China	KP340973	764
17. *P. homoion* (Bychowsky & Nagibina, 1959)	*Rutilus rutilus lacustris* (Pallas, 1814)	Irtysh River (Chinese section), China	KP340972	764
18. *P. diplophyllorchidis* (Jiang et al., 1985)	*Zacco platypus* (Temminck & Schlegel, 1846)	Danjiangkou Reservoir, China	DQ098891	821
19. *P. hemiculteri* (Ling, 1973)	*Hemiculter leucisculus* (Basilewsky, 1855)	Honghu, China	DQ098892	822
20. *P. opsariichthydis* (Jiang *et al*., 1984)	*Opsariichthys uncirostris* (Temminck & Schlegel, 1846)	Danjiangkou Reservoir, China	DQ098890	822
21. *P. parabramisi* (Ling, 1973)	*Parabramis pekinensis* (Basilewsky, 1855)	Tangxun Lake, China	DQ098889	821
22. *P. jiangxiensis* (Jiang et al., 1985)	*Cultrichthys erythropterus* (Basilewsky, 1855)	Tangxun Lake China	DQ098885	822
23. *P. parapeleci* (Jiang *et al*., 1984)	*Parapelecus argenteus* (Günther, 1889)	Danjiangkou Reservoir, China	DQ098882	822
24. *Paradiplozoon yunnanensis* n. sp.	*Sikukia gudgeri* (Smith, 1931)	Lancang River, China	MF775370 *	799

* New sequence obtained in the present study.

## *Paradiplozoon yunnanensis* n. sp.

urn:lsid:zoobank.org:act:57373A86-48E9-4336-B96B-7CCF463B7862

Type host: *Sikukia gudgeri* Smith, 1931 (Teleostei: Cyprinidae). Fishes of the genus *Sikukia* can be distinguished from other fishes of barb by a short snout, large eyes, and lack of an adipose eyelid. *S. gudgeri* is the first species of the genus *Sikukia* to not have barbels and is found only in Thailand and China.

Site of infection: Gills.

Type locality: Jinghong Basin of the Lancang-Mekong River, Yunnan Province, southwest China (22.007777 N, 100.795277 E).

Prevalence of infection: 20% (in total, 12 out of 59 fish).

Intensity of infection: 1–4 with a mean of two parasites per infected fish.

Etymology: The scientific name refers to the name of the province, Yunnan, where the new species was discovered.

Type material: Holotype (X22013.11.13) and four paratypes (M72012.10.29, X32013.11.02, A22013.11.12 and A32013.11.12) deposited at the Section of Zoology, School of Life Sciences of Yunnan Normal University, Kunming, Yunnan Province, China.

### Morphological description

**Figure 1.**
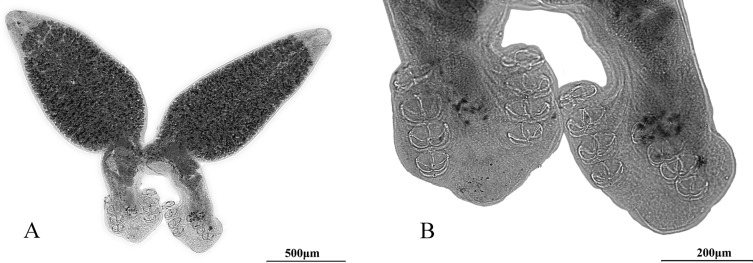
(A) Adult pairs of the parasites fuse in a permanent “X” shape during copulation. (B) Haptor disc-like without a pronounced inflated terminal end.

Adult pairs of the parasites fuse in a permanent “X” shape during copulation (Fig. 1A) with a total body length of 1593 μm (*n* = 10, 1148–2344 μm). Anterior tegument relatively smooth with extremely slight annular transverse folds or plicae. Anterior region approximately 1025 μm long (*n* = 10, 676–1478 μm) and 445 μm wide (*n* = 10, 211–626 μm). Sub-terminal mouth present on the antero-ventral surface, opens into buccal cavity containing two buccal round suckers approximately 46 μm long (*n* = 6, 23–65 μm) and 35 μm wide (*n* = 6, 26–44 μm). Oval, muscular pharynx 45 μm long (*n* = 10, 30–56 μm), 38 μm wide (*n* = 10, 21–52 μm). Pharynx opens into highly branched intestine in the forebody with prominent lateral caeca and distributed vitellaria. Intestine extends from fusion area to hindbody, intestinal caeca pass through reproductive organs and end near attachment apparatus. Haptor disc-like without a pronounced inflated terminal end (Fig. 1B).

Main parts of male and female reproductive organs located in hindbody. Ovary long and strip shaped. Testis, solid and oval-shaped, posterior to the ovary. Eggs oval-shaped, 151 μm long (*n* = 4, 142–160 μm), 75 μm wide (*n* = 4, 74–87 μm), with long curly filament. Follicular vitellaria, well-developed, in fusion region and to the end of hindbody, where the vitelline reservoir forms.

**Figure 2.**
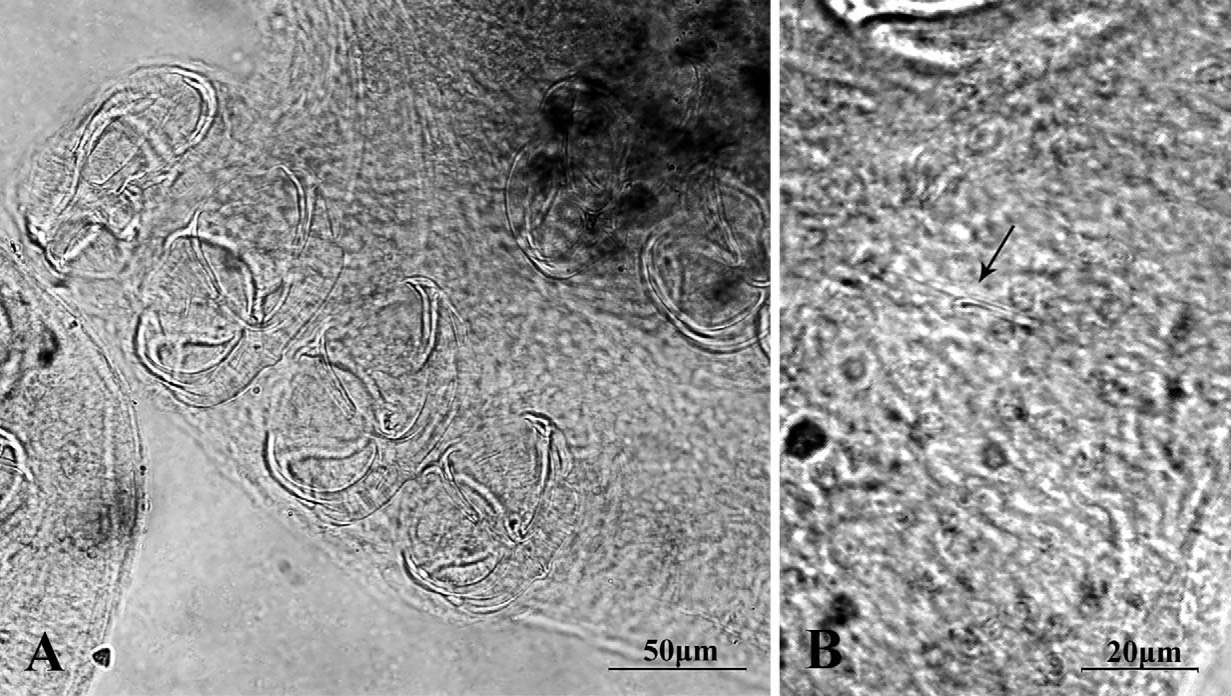
(A) Opisthaptors with four pairs of clamps and (B) a pair of central hooks.

Opisthaptors with four pairs of clamps (Fig. 2A) and a pair of central hooks (Fig. 2B). First clamp 39 μm long (*n* = 9, 18–59 μm), 53 μm wide (*n* = 9, 23–78 μm). Second clamp 44 μm long (*n* = 9, 20–65 μm), 61 μm wide (*n* = 9, 27–86 μm). Third clamp 43 μm long (*n* = 9, 17–61 μm), 62 μm wide (*n* = 9, 29–89 μm). Fourth clamp 46 μm long (*n* = 9, 20–59 μm), 62 μm wide (*n* = 9, 27–87 μm). The first clamp is the smallest, other clamps are similar.

**Figure 3.**
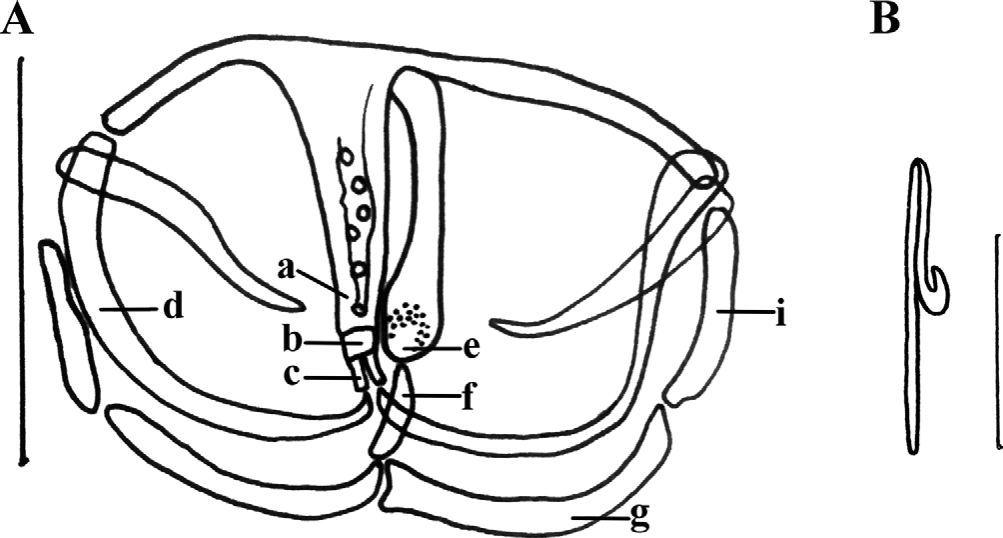
(A) Sclerites of posterior jaw divided into medial parts and lateral parts. (B) Point of the central hooks curls inward.

Clamps formed by slender sclerites. Sclerites of the anterior jaw with slight serrated edge. A longitudinal column of round sclerites in the middle part of the median plate. Anterior end of median plate thickened in marginal area; a narrow rectangular trapeze spur connects to the anterior jaw through two separate anterior joining sclerites. Posterior end of median plate sclerite invaginated. Posterior joining sclerite smooth strip-shaped and longer than the anterior joining sclerites. Sclerites of posterior jaw divided into medial parts and lateral parts (Fig. 3A).

Central hooks located between terminal protrusion of the haptor and first pair of clamps. Length of central hook sickle 12 μm (*n* = 2), handle length 21.5 μm (*n* = 2). Point of the central hooks curls inward (Figs. 2B, 3B).

### Molecular analysis

The obtained final sequence (consensus of three individuals, 100% identity) was 799 bp long (GenBank MF775370). The sequence was analysed with BLAST (base composition). The alignment of ITS2 sequences of 24 taxa was 720 bp in length (including gaps), including 286 conserved sites, 429 variable sites and 349 parsimony informative sites.

Compared with 18 previously submitted sequences of *Paradiplozoon* and five sequences of *Eudiplozoon* (Table 1) to estimate homogeneity by similarity and genetic distance, the obtained rDNA sequence showed similarity to known species, e.g., identity of 89% with *P. hemiculteri* (DQ098892), *P. diplophyllorchidis* (DQ098891), *P. opsariichthydis* (DQ098890), and *P. parabramisi* (DQ098883), and 82% with *P. ichthyoxanthon* (HF566124). The most closely related species to *P. yunnanensis* n. sp. were six species recorded from China (*P. diplophyllorchidis*, *P. opsariichthydis*, *P. parabramisi*, *P. jiangxiensis* (DQ098885), *P. hemiculteri* (DQ098892) and *P. parapeleci*) and two other species (*P. bingolensis* (HE653910) from Turkey and *P. krugerense* (LT574865) from South Africa), with estimated genetic distances (Kimura 2–parameter) of 0.13 and 0.36, respectively (Table 2).

**Table 2.** Pairwise distance (kimura 2-parameter) matrix for the complete ITS2 sequences across all sequences from diplozoid species.

	1	2	3	4	5	6	7	8	9	10	11	12	13	14	15	16	17	18	19	20	21	22	23	24
1		0.0000	0.0024	0.0017	0.0000	0.0266	0.0314	0.0227	0.0211	0.0213	0.0223	0.0214	0.0201	0.0220	0.0299	0.0212	0.0212	0.0341	0.0343	0.0341	0.0339	0.0344	0.0341	0.0365
2	0.0000		0.0024	0.0017	0.0000	0.0266	0.0314	0.0227	0.0211	0.0213	0.0223	0.0214	0.0201	0.0220	0.0299	0.0212	0.0212	0.0341	0.0343	0.0341	0.0339	0.0344	0.0341	0.0365
3	0.0033	0.0033		0.0017	0.0024	0.0270	0.0317	0.0230	0.0214	0.0215	0.0226	0.0217	0.0204	0.0223	0.0303	0.0215	0.0215	0.0342	0.0344	0.0342	0.0340	0.0344	0.0342	0.0369
4	0.0017	0.0017	0.0017		0.0017	0.0268	0.0316	0.0229	0.0213	0.0214	0.0224	0.0215	0.0203	0.0222	0.0301	0.0214	0.0214	0.0344	0.0345	0.0344	0.0341	0.0346	0.0344	0.0368
5	0.0000	0.0000	0.0033	0.0017		0.0266	0.0314	0.0227	0.0211	0.0213	0.0223	0.0214	0.0201	0.0220	0.0299	0.0212	0.0212	0.0341	0.0343	0.0341	0.0339	0.0344	0.0341	0.0365
6	0.2967	0.2967	0.3018	0.2994	0.2967		0.0327	0.0163	0.0202	0.0199	0.0209	0.0200	0.0192	0.0200	0.0309	0.0204	0.0204	0.0367	0.0369	0.0367	0.0370	0.0370	0.0367	0.0368
7	0.3751	0.3751	0.3807	0.3780	0.3751	0.3931		0.0301	0.0291	0.0302	0.0305	0.0299	0.0294	0.0317	0.0136	0.0291	0.0291	0.0288	0.0289	0.0288	0.0289	0.0289	0.0288	0.0301
8	0.2340	0.2340	0.2387	0.2364	0.2340	0.1367	0.3516		0.0145	0.0145	0.0166	0.0149	0.0139	0.0163	0.0281	0.0148	0.0148	0.0319	0.0318	0.0319	0.0321	0.0321	0.0319	0.0327
9	0.2090	0.2090	0.2135	0.2113	0.2090	0.1924	0.3354	0.1128		0.0111	0.0117	0.0110	0.0098	0.0151	0.0269	0.0044	0.0044	0.0320	0.0320	0.0320	0.0322	0.0322	0.0320	0.0343
10	0.2129	0.2129	0.2174	0.2152	0.2129	0.1880	0.3500	0.1114	0.0696		0.0118	0.0063	0.0075	0.0149	0.0281	0.0115	0.0115	0.0330	0.0332	0.0330	0.0332	0.0332	0.0330	0.0351
11	0.2288	0.2288	0.2334	0.2311	0.2288	0.2031	0.3593	0.1408	0.0753	0.0772		0.0117	0.0110	0.0151	0.0287	0.0125	0.0125	0.0331	0.0332	0.0331	0.0333	0.0333	0.0331	0.0347
12	0.2151	0.2151	0.2196	0.2174	0.2151	0.1898	0.3468	0.1172	0.0678	0.0236	0.0753		0.0075	0.0144	0.0275	0.0113	0.0113	0.0332	0.0333	0.0332	0.0334	0.0332	0.0332	0.0348
13	0.1953	0.1953	0.1996	0.1975	0.1953	0.1772	0.3408	0.1036	0.0551	0.0323	0.0680	0.0323		0.0141	0.0272	0.0104	0.0104	0.0324	0.0326	0.0324	0.0326	0.0326	0.0324	0.0332
14	0.2227	0.2227	0.2272	0.2250	0.2227	0.1901	0.3725	0.1369	0.1191	0.1155	0.1192	0.1094	0.1057		0.0294	0.0151	0.0151	0.0334	0.0336	0.0334	0.0336	0.0336	0.0334	0.0353
15	0.3527	0.3527	0.3581	0.3556	0.3527	0.3648	0.0996	0.3216	0.3034	0.3199	0.3318	0.3115	0.3063	0.3408		0.0271	0.0271	0.0285	0.0286	0.0285	0.0287	0.0287	0.0285	0.0297
16	0.2110	0.2110	0.2155	0.2133	0.2110	0.1947	0.3354	0.1168	0.0117	0.0733	0.0847	0.0714	0.0606	0.1191	0.3061		0.0000	0.0318	0.0318	0.0318	0.0320	0.0320	0.0318	0.0345
17	0.2110	0.2110	0.2155	0.2133	0.2110	0.1947	0.3354	0.1168	0.0117	0.0733	0.0847	0.0714	0.0606	0.1191	0.3061	0.0000		0.0318	0.0318	0.0318	0.0320	0.0320	0.0318	0.0345
18	0.4245	0.4245	0.4248	0.4276	0.4245	0.4666	0.3397	0.3914	0.3942	0.4089	0.4113	0.4117	0.3999	0.4169	0.3366	0.3913	0.3913		0.0024	0.0000	0.0017	0.0017	0.0000	0.0158
19	0.4273	0.4273	0.4276	0.4305	0.4273	0.4696	0.3423	0.3913	0.3940	0.4117	0.4141	0.4145	0.4027	0.4198	0.3392	0.3911	0.3911	0.0033		0.0024	0.0029	0.0029	0.0024	0.0158
20	0.4245	0.4245	0.4248	0.4276	0.4245	0.4666	0.3397	0.3914	0.3942	0.4089	0.4113	0.4117	0.3999	0.4169	0.3366	0.3913	0.3913	0.0000	0.0033		0.0017	0.0017	0.0000	0.0158
21	0.4214	0.4214	0.4217	0.4245	0.4214	0.4699	0.3424	0.3944	0.3971	0.4119	0.4143	0.4147	0.4029	0.4200	0.3393	0.3942	0.3942	0.0017	0.0050	0.0017		0.0024	0.0017	0.0160
22	0.4276	0.4276	0.4279	0.4308	0.4276	0.4699	0.3424	0.3944	0.3971	0.4119	0.4143	0.4117	0.4029	0.4200	0.3393	0.3942	0.3942	0.0017	0.0050	0.0017	0.0033		0.0017	0.0160
23	0.4245	0.4245	0.4248	0.4276	0.4245	0.4666	0.3397	0.3914	0.3942	0.4089	0.4113	0.4117	0.3999	0.4169	0.3366	0.3913	0.3913	0.0000	0.0033	0.0000	0.0017	0.0017		0.0158
24	0.4613	0.4613	0.4676	0.4646	0.4613	0.4693	0.3595	0.4033	0.4314	0.4394	0.4377	0.4360	0.4141	0.4450	0.3557	0.4345	0.4345	0.1307	0.1306	0.1307	0.1327	0.1327	0.1307	

Pairwise distances (kimura 2-parameter) between species are shown under the diagonal. Standard error estimates are shown above the diagonal. Appellations of 24 taxa are stated below.1 *Eudiplozoon nipponicum* (AJ300710), 2 *E. nipponicum* (AF369758), 3 *E. nipponicum* (KP340975), 4 *E. nipponicum* (DQ098895), 5 *E. nipponicum* (DQ098895), 6 *Paradiplozoon vaalense* (HG423142), 7 *P. krugerense* (LT574865), 8 *P. ichthyoxanthon* (HF566124), 9 *P. skrjabini* (LC050529), 10 *P. nagibinae* (AJ563371), 11 *P. pavlovskii* (AJ300714), 12 *P. sapae* (AJ300713), 13 *P. bliccae* (AJ300712), 14 *P. megan* (AJ300711), 15 *P. bingolensis* (HE653910), 16 *P. gracile* (KP340973), 17 *P. homoion* (KP340972), 18 *P. diplophyllorchidis* (DQ098891), 19 *P. hemiculteri* (DQ098892), 20 *P. opsariichthydis* (DQ098890), 21 *P. parabramisi* (DQ098889), 22 *P. jiangxiensis* (DQ098885), 23 *P. parapeleci* (DQ098882), 24 *Paradiplozoon yunnanensis* n. sp. (MF775370).

**Figure 4.**
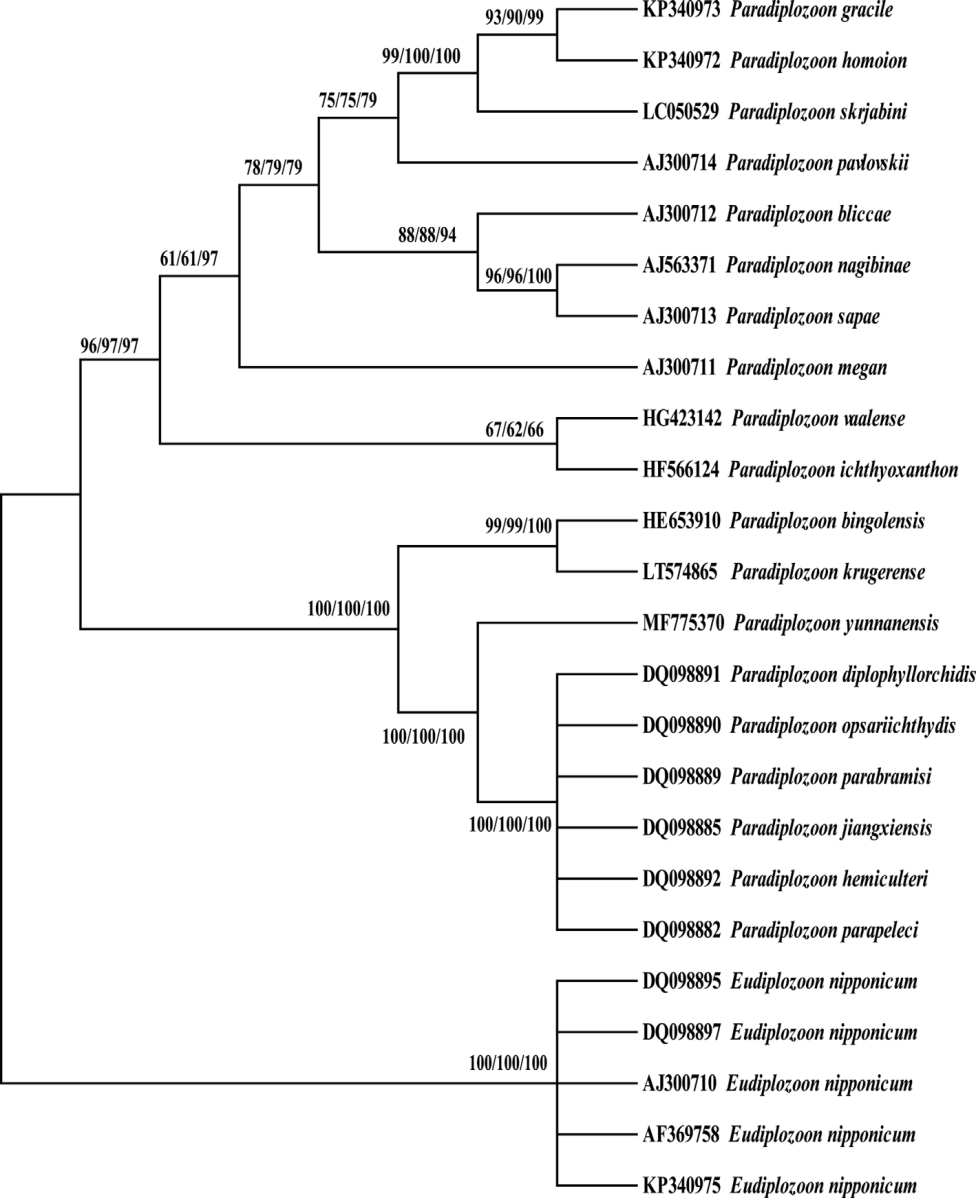
The rooted condensed tree (with 50% cut-off value) based on the NJ/ML/MP analysis method.

According to the rooted condensed tree (with 50% cut-off value) based on the NJ/ML/MP analysis method with *E. nipponicum* as the outgroup (Fig. 4), the new species clustered between two phylogenetic clades: one clade including *P. bingolensis* and *P. krugerense* and another clade with six Chinese species of *Paradiplozoon* species (including *P. diplophyllorchidis*, *P. opsariichthydis*, *P. parabramisi*, *P. jiangxiensis*, *P. hemiculteri* and *P. parapeleci*). The molecular phylogenetic results corroborated morphological taxonomic results and supported *P. yunnanensis* n. sp. as a new taxon.

## Discussion

The Diplozoinae are divided into five genera using the dichotomic keys developed by Khotenovsky [15]. These genera are differentiated based on the presence-absence of dilatation in the middle part of the haptor, the shape and length of lateral branches departing from the intestinal caecum in the posterior end of the body, presence and size of plicae, location of uterine pore, and presence of glandular structures before the suckers [32]. *Paradiplozoon* is the most diverse genus of Diplozoinae and is distinguished from other genera by the absence of a pronounced dilatation in the posterior region of the prehaptor [12, 26]. The posterior part of the specimens in the present study is without tegumental ridges or folds and was not cup-shaped or saucer-shaped. The specimens herein are ascribed to this genus based on the absence of these characters, which is typical of *Paradiplozoon*.

Morphological features (e.g. size of body, size of clamps) vary widely within diplozoid species, depending on size of the host fish and developmental stage of the parasite, making determination to species level difficult [13, 21]. The sclerotized structures such as central hooks and clamp sclerites are considered the structures of most taxonomic relevance for determination of species within the genus *Paradiplozoon*, including the length of the central hook sickle and the shapes of the anterior end of median plate and anterior joining sclerites of the clamps [11, 15, 20–22, 25, 27]. However, the size of the clamps depends on the total length of host fish [8]. The shapes of the trapeze spur and the anterior joining sclerites were used for identifying species [22]. In the present study, the combination of morphological features of clamp and the central hook demonstrates the specimens described herein differ from all other *Paradiplozoon* species.

Monogeneans are monoxenous ectoparasites characterized by a relatively high degree of host specificity, and host specificity has previously been used as a basis for species discrimination. However, *Paradiplozoon homoion* displays low host specificity and infects more than 15 cyprinid host species [22]. *Paradiplozoon yunnanensis* n. sp. is the first and only diplozoid species reported from *S. gudgeri* but this cannot be used as the single argument to demonstrate the validity of the species. Molecular methods based on genetic features of nucleotide sequencing of ITS2 have been suggested as powerful tools for distinguishing species of *Paradiplozoon* [8, 19–22]. Compared with 18 previously submitted sequences of *Paradiplozoon*, the ITS2 sequence of *P. yunnanensis* n. sp. showed 82%–89% similarity to already known species. *Paradiplozoon yunnanensis* n. sp. was most closely related to six Chinese species, *P. bingolensis* and *P. krugerense*, with estimated genetic distances of 0.13 and 0.36 respectively, and was placed in the *Paradiplozoon* clade in the phylogenetic tree. The results combined with morphological characteristics support *P. yunnanensis* n. sp. as a valid species.

Our research provides some information about the phylogenetic status and relationships of 18 species of *Paradiplozoon* by analysing and estimating homogeneity and genetic distance. There are nine species of Chinese *Paradiplozoon* in the reconstructed phylogenetic tree. The Chinese diplozoids *P. gracile* (KP340973) and *P. homoion* (KP340972) clustered in a clade with *P. skrjabini* from Russia (LC050529) firstly, and then with other European diplozoids, including *P. pavlovskii* (AJ300714), *P. bliccae* (AJ300712), *P. nagibinae* (AJ563371), *P. sapae* (AJ300713) and *P. megan* (AJ300711). The NJ topology may be caused by *P. gracile* (KP340973) and *P. homoion* (KP340972) being collected from the Ergis River, Chinese section of the Irtysh River. The Ergis River is the only international river of the Arctic Ocean in China. It originates in the mountains of Altai, northern Xinjiang, China and flows through the Sino Mongolian border [29, 30]. Our results indicate that Chinese diplozoids *P. gracile* (KP340973) and *P. homoion* (KP340972) are very closely related to European diplozoids; this could occur through possible host migration under certain conditions in the Arctic Ocean, which would cause the homogeneity of fish and fish monogeneans.

*Paradiplozoon yunnanensis* n. sp. was confirmed as a new taxon that clustered closely to the clade composed of the other six species of Chinese *Paradiplozoon* with 100% bootstrap support (similarity 82–89%). In addition, our analysis presented significantly non-different genetic distances between the following Chinese species: *P. opsariichthydis, P. parabramisi, P. jiangxiensis, P. hemiculteri*, and *P. parapeleci.* According to Gao et al., the ITS2 sequences of the latter species reached 99% similarity, with interspecific similarity (>99%) between these congeneric species [10]. Only 4 positions were parsimony informative and 13 positions were variable, with 822 conserved positions. Because the BLAST comparison results were the same, Civáňová et al. suggested that the identification of the mentioned Chinese species as different ones was incorrect and strongly suggested their further reclassification [8]. According to Jirsová et al., related sequences available in GenBank are probably misnamed as *P. hemiculteri* [13]. Furthermore, morphological characteristics may be variable within species and congeneric species. Therefore, we agree that morphological differences and host species are not the most suitable taxonomical features for identifying congeners. On the other hand, the geographical distribution of hosts plays an important role in the occurrence and host-specificity of diplozoids. According to Reichenbach-Klinke, *Diplozoon homoion* Bychowsky and Nagibina is predominant in Central Europe and is probably divided into subspecies [24]. Taxonomic studies of Chinese *Paradiplozoon* diplozoids are necessary. It is worthy of further study to supplement molecular information of Chinese recorded *Paradiplozoon* to provide suitable indicators for precluding morphological resemblance among these species.

The Lancang-Mekong River is an international river in Asia. It flows through China, Myanmar, Laos, Thailand, Cambodia and Vietnam, and is called Lancang River in southwest China, Yunnan Province. The geographical position of the river is responsible for its unique biological diversity [14]. There are 13 orders, 42 families, 198 genera and 620 valid fish species recorded in Yunnan Province. Of these fishes, 586 species are native species, 34 are alien species, and 254 are endemic to Yunnan, with more than 300 species of the family Cyprinidae and 152 species occurring within China only in Yunnan Province. The freshwater fish fauna in the Lancang River (upper Mekong) has a distinctly rich biodiversity, with 12 subfamilies, 105 genera and 183 species [6]. Although diplozoids are generally considered parasites of cyprinid species, only 36 species have been recorded in China; some species require further study. Cyprinids are currently the only recorded fish family hosting diplozoids in south-eastern Asia [18, 21]. Description of new taxa and molecular phylogeny will contribute to a clearer understanding of the mechanism of historical biogeography and coevolution of Monogenea-fish associations in south-eastern Asia.

## Conflict of interest

All authors have no conflict of interest. We acted in accordance with all applicable institutional and national laws and guidelines during this research.
